# Enablers of Physician Prescription of a Long-Term Asthma Controller in Patients with Persistent Asthma

**DOI:** 10.1155/2016/4169010

**Published:** 2016-06-15

**Authors:** Francine M. Ducharme, Alexandrine J. Lamontagne, Lucie Blais, Roland Grad, Kim L. Lavoie, Simon L. Bacon, Martha L. McKinney, Eve Desplats, Pierre Ernst

**Affiliations:** ^1^Department of Pediatrics, University of Montreal, Montreal, QC, Canada H3T 1C5; ^2^Clinical Research and Knowledge Transfer Unit on Childhood Asthma, Research Centre, CHU Sainte-Justine, Montreal, QC, Canada H3T 1C5; ^3^Department of Social and Preventive Medicine, University of Montreal, Montreal, QC, Canada H3C 3J7; ^4^Department of Pharmacology, University of Montreal, Montreal, QC, Canada H3T 1J4; ^5^Department of Family Medicine, McGill University, Montreal, QC, Canada H3T 1E2; ^6^Montreal Behavioural Medicine Centre, Hôpital du Sacré-Cœur de Montreal, Montreal, QC, Canada H4J 1C5; ^7^Department of Psychology, Université du Québec à Montreal, Montreal, QC, Canada H3C 3P8; ^8^Department of Exercise Science, Concordia University, Montreal, QC, Canada H4B 1R6; ^9^Research Centre, CHU Sainte-Justine, Montreal, QC, Canada H3T 1C5; ^10^Divisions of Clinical Epidemiology and of Pulmonary Medicine, Department of Medicine, Jewish General Hospital, Montreal, QC, Canada H3T 1E2; ^11^Department of Medicine, McGill University, Montreal, QC, Canada H4A 3J1

## Abstract

*Objective*. We aimed to identify key enablers of physician prescription of a long-term controller in patients with persistent asthma.* Methods*. We conducted a mailed survey of randomly selected Quebec physicians. We sent a 102-item questionnaire, seeking reported management regarding one of 4 clinical vignettes of a poorly controlled adult or child and endorsement of enablers to prescribe long-term controllers.* Results*. With a 56% participation rate, 421 physicians participated. Most (86%) would prescribe a long-term controller (predominantly inhaled corticosteroids, ICS) to the patient in their clinical vignette. Determinants of intention were the recognition of persistent symptoms (OR 2.67), goal of achieving long-term control (OR 5.31), and high comfort level in initiating long-term ICS (OR 2.33). Decision tools, pharmacy reports, reminders, and specific training were strongly endorsed by ≥60% physicians to support optimal management. Physicians strongly endorsed asthma education, lung function testing, specialist opinion, accessible asthma clinic, and paramedical healthcare professionals to guide patients, as enablers to improve patient adherence to and physicians' comfort with long-term ICS.* Interpretation*. Tools and training to improve physician knowledge, skills, and perception towards long-term ICS and resources that increase patient adherence and physician comfort to facilitate long-term ICS prescription should be considered as targets for implementation.

## 1. Introduction

Guided self-management is the cornerstone of the management of adults and children with asthma [1–4]. Five evidence-based recommendations for guided self-management were endorsed in national and international asthma guidelines: prescription of long-term controller medication for those with persistent asthma; provision of self-management plans; regular medical review; environmental control of known triggers; and asthma education [1–3, 5]. Suboptimal management results in frequent exacerbations, preventable hospitalizations, unsafe use and abuse of medications, absenteeism, and even death [6–8]. Yet, fewer than 30% of patients with persistent asthma use daily controller medications or have a written self-management plan [9, 10]. Although some of the responsibilities lie with the patient, physicians are also at fault as fewer than half of physicians report basing their treatment recommendations on the national asthma guidelines [9, 11, 12]. Barely two-thirds of physicians self-report recommending daily inhaled corticosteroids (ICS) in patients with persistent asthma [10], and most physicians prescribe only short courses of ICS [13–15] or provide insufficient prescription renewals to allow long-term use of ICS [16, 17].

Several studies have addressed barriers to physician adherence to asthma guidelines including use of asthma controllers [18–22], but few, if any, specifically addressed long-term therapy. Yet, the identification of relevant barriers is insufficient by itself to design an effective intervention. Indeed, many knowledge translation initiatives were unsuccessful or only modestly effective because the tested intervention did not address the relevant barriers or lacked a foundation in behavioural theory supporting the intervention [23, 24]. Thus, one must ensure that solutions are effective and implementable by the target audience. Seeking physicians' enablers and proposed solutions has emerged as an effective approach to identify such interventions [25]. In a recent qualitative study, we identified a large number of enablers proposed by physicians to optimise asthma care [26]. Marked variation across physician specialties in prescription patterns and reported barriers suggest the need to target interventions to specific settings and/or specialties [18, 19, 22, 27]. High endorsement of promising enablers by physicians and identification of operational behavioural targets are thus keys to designing a successful intervention to improve their practice [24, 28, 29].

Our main objective was to quantify physicians' endorsement of promising enablers to facilitate the prescription of long-term ICS. We also wished to ascertain physician-reported behaviour regarding the prescription of long-term asthma controller in a poorly controlled patient with persistent asthma and the determinants of this behaviour.

## 2. Methods

### 2.1. Study Design

The current paper reports the survey of randomly selected Quebec physicians treating patients with asthma. The protocol received approval from the Institutional Review Board of the Sainte-Justine University Health Centre. All participants received an information letter and consent was assumed if they returned the completed questionnaire.

### 2.2. Participants

Physicians were eligible if they were registered in July 2013 with the* College des Médecins du Québec* as family medicine physicians, pediatricians, or emergency physicians and held an active practice licence. Physicians were excluded if they had obtained their diploma more than 30 years earlier, were not practicing, were not seeing patients with asthma, were in training, or had participated in the questionnaire pretest; the former criteria was meant to exclude physicians most likely to be retired by the time a specific knowledge translation intervention would be ready for testing. Physicians were randomly selected using a stratified sampling procedure based on specialty.

### 2.3. Materials

#### 2.3.1. Item Generation

In the first phase of the study [26] where we conducted qualitative semistructured interviews of 42 physicians, we identified 867 enablers of optimal guided self-management and specifically of the prescription of long-term ICS.

#### 2.3.2. Item Reduction

We retained enablers most frequently endorsed by interviewed physicians; among those, a 2-step Delphi approach was conducted among 7 coauthors with expertise in pediatric and adult respirology, family medicine, pediatrics, pharmacoepidemiology, and behaviour change to identify enablers most likely to be implementable.

#### 2.3.3. Presentation and Scaling

The self-report questionnaire had five main sections. The first section served to describe the characteristics and practice setting of physicians. In the second section, participants were asked to select one of 4 clinical vignettes that most closely reflected their practice, namely, a school-aged child or an adult with poorly controlled asthma who presented either for an acute exacerbation or in the clinic setting when stable (Table E1 in Supplementary Material available online at http://dx.doi.org/10.1155/2016/4169010). Based on the selected vignette, participants were asked to report their treatment recommendations and follow-up strategy (i.e., behaviour), assessment of asthma control and phenotype (i.e., knowledge and skills), and treatment objectives (i.e., goals). The third section pertains to physicians' comfort level in performing key tasks associated with optimal asthma management (i.e., confidence about capabilities), perceived risk-benefit associated with prescription of long-term ICS for certain groups of patients (i.e., knowledge of risk-benefit), and endorsement of resources to assist in patient-specific decision-making and/or patient's adherence to ICS. The fourth and fifth sections, pertaining to written action plans and pharmacists' professional activities, are the object of other reports. Responses were recorded on a 6-point Likert scale ranging from 0 to 5.

#### 2.3.4. Pretesting for Clarity

Pretested in six physicians, the questionnaire took between 20 and 30 minutes to be complete. It was endorsed by and included the logos of the* Institut National d'Excellence en Santé et Services Sociaux* (INESSS),* Association des Pédiatres du Québec*,* Association des Spécialistes en Médecine d'Urgence du Québec,* and* Fédération des Médecins Omnipraticiens du Québec.*

#### 2.3.5. Survey Procedures

Using the Tailored Design Methods [30], a prenotification postcard was sent, followed 10 days later by the information letter, questionnaire, and a $25 cheque, a thank you/reminder postcard on day 21, and, for nonresponders, a second questionnaire on day 37, followed by up to three phone calls. Where feasible, another physician was selected to replace those identified as ineligible (by phone or questionnaire). The deadline for returning completed questionnaires was April 2014.

### 2.4. Statistical Analysis

A sample size of 500 physicians was required to obtain a precision of ±5% for endorsement proportions of 50%. Assuming a 60% response rate, the questionnaire was sent to 838 physicians.

The distribution of endorsement was presented as median (25%, 75%), after adjustment for the stratified sampling of physicians by specialty (91.0% for family physicians, 7.6% pediatricians, and 1.4% emergency physicians); we illustrated key results with diverging stacked bar charts [31]. We classified physicians as “intenders” or “nonintenders” based on their reported behaviour on the selected clinical vignette to prescribe long-term asthma controller for at least 3 months or until the patient sees his/her treating physician. Physicians were deemed to be in “strong agreement” if they responded 4 or 5 on the Likert-like scale of 0 to 5. We explored the determinants of physicians' intention to prescribe long-term ICS, using bivariate logistic regression analyses to identify those significantly associated with the outcome; these were offered as candidate variables in the multivariate logistic regression analysis, forcing medical specialty in the model. Potential determinants included the following: physician characteristics; selected vignette; assessment of control; treatment goals; comfort level with initiating long-term ICS; and level of hesitation about risk-benefit of long-term ICS in various patients. All tests were two-sided with estimates presented with 95% confidence intervals. Analyses were performed on SAS® 9.3 software (SAS Institute Inc., Cary, NC 27513, USA). *P* values less than 0.05 indicated statistical significance, with no correction for multiple testing and no imputation for missing data.

## 3. Results

**Figure 1:**
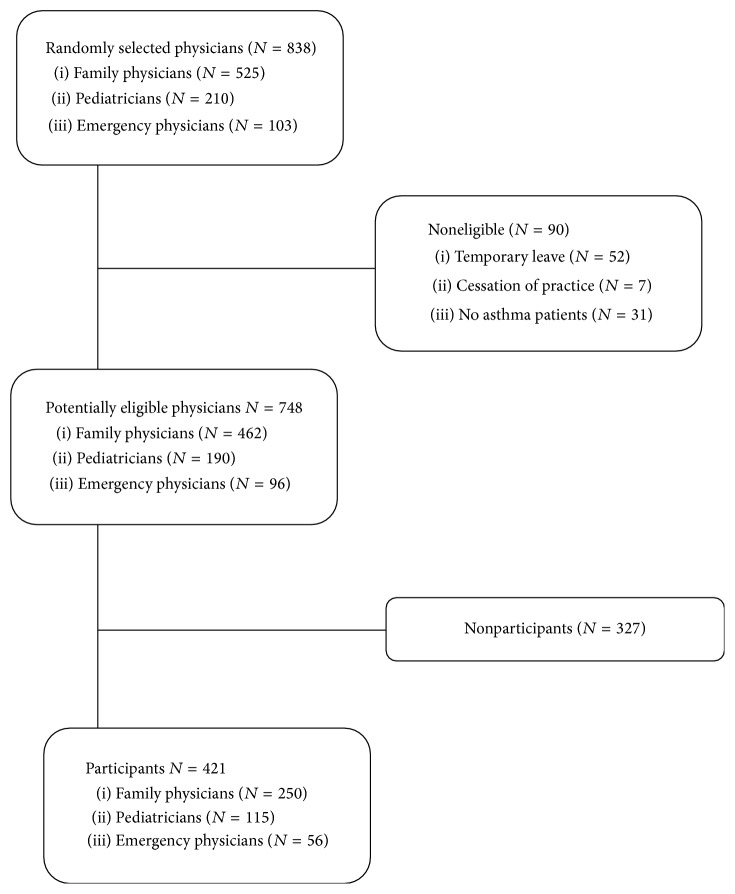
The flow of participants is depicted from screening to analysis.

The survey was sent to 838 physicians: 525 family physicians, 210 pediatricians, and 103 emergency physicians. After excluding 90 (10.7%) noneligible physicians, 421 (56%) of 748 potentially eligible physicians returned the completed questionnaire (Figure 1). Nonrespondents were similar to the respondents in specialty and practice area but had been in practice for a median of 7 years longer with a higher proportion of males (Table E2). Participants were predominantly women (69%), working in an urban environment (93%) and nonacademic institution (44%); patients with asthma represented about a quarter of their clientele (Table 1).

Approximately 60% of participants selected the acute-care vignettes, with the remainder, the clinic vignettes, equally distributed between the pediatric and adult cases. Characteristics of respondents selecting each clinical vignette, their assessment, and intended prescription are displayed in Table E3. Although nearly all physicians recognised the suboptimal asthma control, about a quarter of respondents perceived the patient as having intermittent symptoms. The overwhelming majority (94.2%) would prescribe an asthma controller, usually ICS, as monotherapy or combination therapy, and 86.0% would prescribe the controller for long-term use. Short-term treatment objectives were sought by most, particularly in acute-care vignettes, yet the overwhelming majority of physicians also reported long-term treatment goals.

Most physicians reported being comfortable with diagnosing asthma, distinguishing between intermittent and persistent asthma, assessing asthma control, and initiating long-term ICS, and reported low hesitation regarding the risk-benefit ratio of prescribing long-term ICS in the age group selected in the vignette. However, the overall comfort level was low for distinguishing between intermittent and persistent asthma without lung function tests, with significant hesitation regarding the risk-benefit ratio of prescribing long-term ICS in patients with intermittent or mild asthma (Table E4).

The three most important determinants of the intention to prescribe a long-term controller, after adjustment for speciality, were the following: the physician's goal of achieving long-term asthma control, recognition of symptoms as being persistent, and comfort level in initiating a therapy of long-term ICS (Table 2). When the comfort level was removed as a candidate variable, it was replaced by low hesitation level regarding the risk-benefit of prescribing long-term ICS (Table E5).

**Figure 2:**
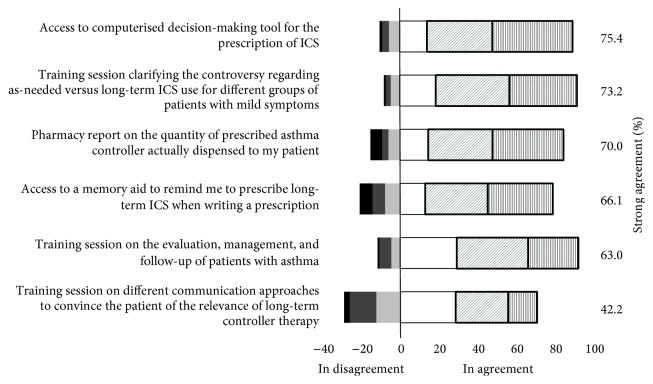
This histogram depicts the physicians' endorsement, adjusted for the sampling fraction, of each proposed enablers on a Likert-like scale ranging between 5 indicating strong agreement (vertical bars), 4 (diagonal grey bars), 3 (white), 2 (light grey), 1 (medium grey), and 0 indicating strong disagreement (black). The proportion of participants with strong endorsement, that is, answering 4 or 5, is identified by a dark box in the histogram and displayed in the right column.

The six enablers, previously identified by physicians to support their general management approach regarding long-term ICS use [26], are depicted in Figure 2. To increase their confidence in both the patient-specific indication for, and patient adherence to, long-term ICS, physicians strongly endorsed several resources, namely, patient asthma education, lung function tests, concordant recommendation by a specialist, and shared responsibility with paramedical healthcare professionals (nurses, certified asthma educators, pharmacists, and respiratory technicians) (Table 3). Most respondents did not know the expected delay to access these resources; when known, significant median delays were reported particularly for lung function testing (1–3 months) and consultation with an asthma specialist (≥4 months) (Figure E1). There was a strong interest in having a computerised system to identify delays to these resources in various areas and to be informed of novelties in asthma management (96.1%), primarily by training days, distance online learning, and application for tablets/smartphones.

## 4. Interpretation

In this group of randomly selected Quebec physicians, most reported that they would prescribe a long-term asthma controller, predominantly ICS, to the poorly controlled patient depicted in their selected vignette. Physicians highly endorsed training and tools to support their general management approach. With regard to patient-specific decision-making, key enablers to improve their comfort level in prescribing, and perceived patient adherence to, long-term ICS included the following: patient asthma education, lung function testing, concordant opinion by a specialist, having access to an asthma clinic to refer patients, and paramedical healthcare professionals to assist in guided self-management. The most important features distinguishing physicians who would prescribe a long-term asthma controller were the recognition of symptoms as being persistent, their high comfort level in initiating long-term ICS, and their goal of improving long-term asthma control. The substitution of comfort level in, by less hesitation regarding the risk-benefit ratio of, prescribing long-term ICS suggested that the latter is inversely and closely related to the former.

The strong endorsement of specific training sessions, decision-support tools, reminders, and pharmacy reports of drug claims to support the general management approach is aligned with prior studies [32]. In these studies, physicians voiced, as barriers to optimal asthma management, their confidence about capabilities in prescribing, and beliefs about consequences of, long-term ICS, as well as their worry about patients' noncompliance and the absence of patient follow-up [10, 33, 34]. In addition, key resources that increase physician's reported comfort in prescribing long-term ICS and perceived patient adherence were endorsed for patient-specific management, presumably because of more certainty in the management decision (i.e., lung function testing, specialist's opinion, and access to an asthma clinic), greater degree of collaborative care and patient follow-up (i.e., patient guidance and education provided by a paramedical healthcare professional) [33]. A highly valued proposal was to provide computerised systems to identify the delay for access to these resources, a solution implemented with success for emergency wait time [35]. Most strongly endorsed enablers have been shown to be effective to improve physicians' prescription in general, and of asthma controllers specifically, namely, facilitated workshops (by improving knowledge, attitude, skills, and beliefs) [36], decision-support tools [32] and, to a lesser extent, reminders [37], and organisational changes [32].

With over four-fifths prescribing long-term asthma controller to their patient in the clinical vignettes, the prescription behaviour was highly concordant with recent national and international guidelines [1, 4, 38]. Yet, it contrasted with published prescription patterns varying between 17% and 69% of patients receiving ICS in Quebec and elsewhere [10, 15, 39], often with inadequate number of renewals to enable long-term use [15, 16]. The latter suggests more emphasis on physician's short-term treatment goals and/or suboptimal prescription filling by patients [39]. Although reported prescribing behaviour may overestimate real practice patterns, the apparent discrepancy with prescriptions studies may be due in part to the fact that poorly controlled patients as described in the vignettes may represent a small proportion of patients enrolled in drug claim data; alternatively it may reflect evolving practice patterns [40].

In contrast to prior reports indicating the lowest use of long-term asthma controller by emergency physicians compared to family physicians and other specialists [10, 27, 33, 39], specialty or practice setting was not important in the multivariate analysis. Indeed, physicians' perceived patient need for ICS, treatment goals, confidence about capabilities in prescribing, and/or knowledge of risk-benefit regarding long-term ICS, all recognised domains for effective implementation of any health behaviour, appear to be the key operational constructs in the prescription of long-term ICS [27]. This is in line with prior reports, in which confidence about capabilities was associated with an 2.8 OR of prescribing ICS [10]. The strength of associations and consistency of predictors across age groups in our vignettes underline the robustness of the predictors of intention.

Despite a large sample size and the strength and precision of identified associations, we acknowledge the following study limitations. Consistent with prior physician surveys, there was a slight overrepresentation of female physicians and, importantly, those in practice for a shorter period [41]; findings may thus reflect more the practice of physicians trained under recent guidelines than those trained when guidelines and teaching methods were different. We acknowledge the possibility of a social desirability bias, that is, the tendency for physicians to report the perceived desired response rather than their true behaviour, which would overestimate actual use of ICS. To minimise such bias, we provided a large range of response options for management questions in vignettes and reversed questions, asking first about management and last about patient assessment and treatment goals. Recent data showing concordance in determinants of prescription of ICS, such as those observed in this study, suggest generalizability [10].

The study was conducted in Quebec where there is free access to medical care, free patient asthma education, and a subsidised drug plan for residents. Despite our long questionnaire, our 56% response rate (59%, assuming that 11% of nonresponders were ineligible as noted among those reached) is within expected standards (54%–60%) for physician surveys, in which higher response rates are generally not associated with less bias [41]. Our objective to have a representative sample of physicians treating patients with asthma resulted in a large proportion of respondents being family physicians. Their frequent selection of the acute-care and pediatric vignettes suggests that an important proportion of these patients are indeed treated by family physicians in Quebec, which may not be applicable to other countries. Caution is thus advised before generalization of the study results to other healthcare settings or specialties.

The overwhelming majority of surveyed physicians reported prescribing long-term ICS to the poorly controlled patient in the clinical vignette, attesting to their intention. In line with the observed determinants of intention, training sessions and decision-support tools to improve physician recognition of persistent symptoms, the importance of long-term asthma control, and confidence in, or knowledge about the risk-benefits of, initiating long-term ICS carry the best chance of improving the rate of long-term ICS prescriptions in patients with persistent asthma. Enhancing access to key resources to support patient-specific management decisions appears to be crucial enablers to optimal asthma management and may lead to greater patient adherence. The physician-endorsed strong enablers provide important insights into design promising implementation interventions to improve long-term ICS prescription.

## Supplementary Material

The supplemental materiel describes the clinical vignettes (Table E1), the comparison between participants and non-participants (Table E2), the reported assessment of, and prescription for, each case vignette (Table E3), the beliefs about capabilities and consequences regarding the prescription of long-term asthma controller (Table E4), the multivariate analysis of physician's intention of prescribing long-term asthma controller (Table E5) and the reported access to each resource (Figure E1).

## Figures and Tables

**Table 1:** Characteristics of respondents.

	Participants (*N* = 421)
Male sex, *n* (%)	131 (31)
Years in practice, median (25%, 75%)	13 (5, 21)
Speciality, *n* (%)	
Family medicine	250 (60)
Pediatrics	115 (27)
Emergency medicine	56 (13)
Primary practice location, *n* (%)	
Urban	390 (93)
Rural	31 (7)
Completed training, *n* (%)^*∗*^	
Family medicine (residency)	272 (65)
Pediatrics (residency or fellowship)	121 (29)
Respirology (residency or fellowship)	117 (28)
Emergency medicine (residency or fellowship)	69 (16)
Other (residency or fellowship)	55 (13)
Practice setting, *n* (%)	
Clinic with appointment	285 (68)
Walk-in clinic	168 (40)
Emergency room	168 (40)
Intensive care unit	26 (6)
Hospital wards	171 (41)
Home care	40 (10)
Others	77 (18)
Proportion of clientele with asthma, median (25%, 75%)	27 (18, 27)
Proportion of children in clientele, median (25%, 75%)	55 (9, 82)
Practice in an asthma clinic, *n* (%)	13 (3)
Self-reported being an asthma specialist, *n* (%)	50 (12)
Usual work environment, *n* (%)	
Academic institution	185 (44)
Nonacademic institution	48 (11)
Private, group, or community practice	187 (45)

^*∗*^The training completed was not mutually exclusive. Indeed, several physicians reported two or more training programs such as family medicine (or pediatric) with emergency medicine, a popular training to serve as general (or pediatric) emergency physicians.

**Table 2:** Multivariate analysis of intention of prescribing long-term asthma controller.

	Intenders^¶^ (*N* = 338)	Nonintenders^¶^ (*N* = 82)	All cases	Pediatric case vignettes	Adult case vignettes
			Odd ratios^∫ ^ (95% CI)	Odd ratios^∫ ^ (95% CI)	Odd ratios^∫ ^ (95% CI)
*Types of symptoms* ^**∗**^, *n* (%)					
Persistent	281 (83.1)	49 (59.8)	2.67 (1.54, 4.63)		2.40 (1.44, 5.02)
*Treatment objective* ^**∗**^, *n* (%)					
Improving long-term control	298 (88.4)	42 (51.2)	5.31 (2.74, 10.3)		7.56 (2.99, 19.28)
*Level of comfort* ^†^, median (25%, 75%)					
Initiating long-term inhaled corticosteroids	4.0 (1.0, 5.0)	3.0 (1.0, 5.0)	2.33 (1.67, 3.24)	5.98 (3.00, 11.92)	1.50 (1.02, 2.21)
*Specialty*, *n* (%)					
Pediatrics	104 (30.8)	11 (13.4)	0.87 (0.43, 1.77)	0.59 (0.24, 1.43)	
Emergency medicine	31 (9.2)	25 (30.5)	0.81 (0.43, 1.54)	0.91 (0.32, 2.62)	0.64 (0.28, 1.46)
Family medicine	203 (60.1)	46 (56.1)	1	1	1

Blank cells indicate that the variable was not statistically significant.

^¶^Physicians who reported prescribing long-term ICS to the patient in their selected vignette were considered “intenders” in contrast to their counterparts, considered “nonintenders.”

^*∗*^Regarding the patient in their selected case vignette.

^†^On a Likert scale of 0 (not comfortable at all) to 5 (very comfortable).

^∫ ^Odds ratio adjusted for speciality.

**Table 3:** Resources that support physician's prescription of, and patient's compliance to, long-term inhaled corticosteroids (ICS).

Enablers	↑ physician's comfort to prescribe long-term ICS	↑ patient adherence to long-term ICS	Access to service^§^	Interest in computerised system to identify access delay
	Adjusted proportion^*∗*^ (95% CI)	Adjusted proportion^*∗*^ (95% CI)	Adjusted proportion^†^ (95% CI)	Adjusted proportion^*∗*^ (95% CI)
Patient's asthma education	65 (59, 71)	95 (93, 98)	86 (80, 90)	67 (62, 73)
Finding the closest asthma education centre	—	—	—	81 (77, 86)
Lung function tests for school-aged children/adults	70 (64, 76)	71 (65, 76)^‡^	97 (95, 99)	70 (65, 76)
Lung function tests for preschoolers	68 (62, 74)		47 (38, 56)	
Concurrent opinion from a specialist	71 (65, 77)	62 (57, 66)	96 (93, 98)	70 (65, 76)
Frequent follow-up visits	—	66 (60, 71)	—	—
Asthma clinic to refer patients	78 (73, 83)	—	60 (53, 67)	71 (65, 72)
Paramedical healthcare professional^¶^				
To guide patient in the treatment plan	78 (73, 83)	92 (88, 95)	—	—
Available on site to provide asthma education	76 (70, 81)	—	52 (46, 58)	—
To share patient follow-up	—	—	57 (50, 63)	—

^*∗*^Values are reported as “*adjusted proportion*” of high endorsement, that is, 4 or 5 on the Likert scale, after adjustment for the stratified sampling of physicians by specialty, that is, weighting responses to reflect the distribution of physicians in the Province of Quebec using weights of 91.0% for family physicians, 7.6% for pediatricians, and 1.4% for emergency physicians.

^†^Values are reported as “*adjusted proportion*” of those that declared access, adjusted for the stratified sampling of physicians by specialty, as described above by^*∗*^.

^‡^Lung function testing for any age group.

^¶^Including nurses, certified asthma educators, pharmacists, and respiratory technicians.

## Abbreviations

ICS: Inhaled corticosteroids.
