# Supplementary material for: Enablers of Physician Prescription of a Long-Term Asthma Controller in Patients with Persistent Asthma
Source: Can Respir J. 2016 Jun 15;2016:4169010. doi: 10.1155/2016/4169010 (PMC4925971; doi:10.1155/2016/4169010)
Supplement: Supplementary file 1 — The supplemental materiel describes the clinical vignettes (Table E1), the comparison between participants and non-participants (Table E2), the reported assessment of, and prescription for, each case vignette (Table E3), the beliefs about capabilities and consequences regarding the prescription of long-term asthma controller (Table E4), the multivariate analysis of physician's intention of prescribing long-term asthma controller (Table E5) and the reported access to each resource (Figure E1). [file 4169010.f1.pdf]

Supplemental materiel

Enablers of physician prescription of a long-term asthma controller in patients with persistent asthma

Francine M. Ducharme, M.D., M.Sc., Alexandrine J. Lamontagne, Lucie Blais, Ph.D, Roland Grad, M.D., CM, M.Sc,, Kim L. Lavoie, Ph.D., Simon L. Bacon, Ph.D., Martha L. McKinney, MD, M.Ph., FRCP(c), Eve Desplats, Ph.D, Pierre Ernst M.D., M.Sc..

Table E1. Clinical Vignettes

Table E2. Comparison of participants and non-participants

Table E3. Reported assessment of, and prescription for, each case vignette

Table E4. Beliefs about capabilities and consequences regarding the prescription of long-term asthma controller

Table E5. Multivariate analysis of physicians' intention of prescribing long-term asthma controller

Figure E1. Reported access to each resource

Table E1. Clinical Vignettes

Setting	Child	Adult
	Acute exacerbation	Acute exacerbation
Walk-in clinic, Emergency Department or hospital inpatient unit	Alex is a **seven year old boy** who presents to the walk-in clinic, emergency room or hospital inpatient unit in which you work. This is his second medical visit in the past six months for an asthma exacerbation triggered by a common cold. As usual, he started his inhaled corticosteroids and salbutamol at the onset of the cold, two days ago. He now presents with rapid breathing, decreased breath sounds bilaterally and scattered expiratory wheezing. There is no fever and no suspicion of other complicating conditions. Usually, he coughs for two to three weeks with a cold, particularly at night, which has led him to miss a week of **school** in the past 6 months. Between colds, he takes no regular asthma therapy other than his blue inhaler, taken about four times per week, for shortness of breath or wheezing. Alex has not seen his treating physician in over a year. Although he presented with a moderate asthma exacerbation, he has now responded satisfactorily to one dose of oral corticosteroids and repeated doses of inhaled salbutamol. He is now sufficiently well to go home.	Alex is a **35-year old man** who presents to the walk-in clinic, emergency room or hospital inpatient unit in which you work. This is the second medical visit in the past six months for an asthma exacerbation triggered by a common cold. As usual, he started his inhaled corticosteroids and salbutamol at the onset of the cold, two days ago. He now presents with rapid breathing, decreased breath sounds bilaterally and scattered expiratory wheezing. There is no fever and no suspicion of other complicating conditions. Usually, he coughs for two to three weeks with a cold, particularly at night, which has led him to miss a week of **work** in the past six months. Between colds, he takes no regular asthma therapy other than his blue inhaler, taken about four times per week, for shortness of breath or wheezing. Alex has not seen his treating physician in over a year. Although he presented with a moderate asthma exacerbation, he has now responded satisfactorily to one dose of oral corticosteroids and repeated doses of inhaled salbutamol. He is now sufficiently well to go home.
	1-month after acute exacerbation	1-month after acute exacerbation
Office or private practice - visit by appointment	Alex is a **seven year old boy** whom you are seeing by appointment for the first time for asthma in your office or private practice, following a visit to an emergency room four weeks ago. **It was his second** acute-care **visit** in the past six months for an asthma exacerbation triggered by a common cold. As usual, he had started his inhaled corticosteroids and salbutamol at the onset of his cold. On discharge from the emergency room, he was given a five-day course of oral corticosteroids, inhaled salbutamol for use as needed, and a four-week course of inhaled corticosteroids. Alex has not seen his treating physician in over a year. He reports no daytime or nighttime symptoms and no use of salbutamol in the past week. He reports being a bit short of breath during exercise. This is a marked improvement from the past six months. During that time, even between colds, he was bothered at night with cough about once per week and used his blue inhaler three to four times per week for shortness of breath or wheezing. **His parents** stopped his inhaled corticosteroids one week ago because he felt well.	Alex is a 35-year-old man whom you are seeing by appointment for the first time for asthma in your office or private practice, following a visit to an emergency room four weeks ago. **He has had** two acute-care **visits** in the past six months for an asthma exacerbation triggered by a common cold. As usual, he had started inhaled corticosteroids and salbutamol at the onset of his cold. On discharge from the emergency room, he was given a five-day course of oral corticosteroids, inhaled salbutamol for use as needed, and a four-week course of inhaled corticosteroids. Alex has not seen his treating physician in over a year. He reports no daytime or nighttime symptoms and no use of salbutamol in the past week. He reports being a bit short of breath during exercise. This is a marked improvement from the previous six months. During that time, even between colds, he was bothered at night with cough once per week and used his blue inhaler about three to four times per week for shortness of breath or wheezing. **He** stopped his inhaled corticosteroids one week ago because he felt well.

Table E2. Comparison of participants and non-participants

	Participants (N = 421)	Non-participants (N = 327)	*p*-value
Male sex - n (%)	131 (31)	142 (43)	< 0.001
Years in practice - Median (25%, 75%)	13 (5, 21)	20 (5, 25)	< 0.001
Practice area - n (%)			0.17
Urban	390 (93)	311 (95)	
Rural	31 (7)	16 (5)	
Speciality - n (%)			0.30
Family medicine	250 (60)	212 (65)	
Pediatrics	115 (27)	75 (23)	
Emergency Medicine	56 (13)	40 (12)	

Table E3. Reported assessment of, and prescription for, each case vignette

	Child		Adult		All vignettes
	**Acute exacerbation N=123**	**1 month after exacerbation N=89**	**Acute exacerbation N=128**	**1 month after exacerbation N=80**	**Adjusted proportion ‡ (95% CI)**
**Male sex** - n (%)	28 (22.8)	16 (17.9)	61 (47.7)	26 (32.5)	27.9 (22.5 - 33.3)
**Speciality** - n (%)					
Family medicine	38 (30.9)	41 (46.1)	91 (71.1)	79 (98.8)	96.1 (95.3 - 96.9)
Pediatrics	67 (54.5)	48 (53.9)	0 (0)	0 (0)	3.5 (2.8 - 4.3)
Emergency Medicine	18 (14.6)	0 (0)	37 (28.9)	1 (1.2)	0.4 (0.3 - 0.5)
**Years of practice** -median (25%, 75%)	10 (4, 18)	14 (4, 22)	12 (5, 21)	20 (8, 27)	12.1 (4.0, 20.9)
**Assessment of asthma control - n (%)**					
Well controlled	1 (0.8)	0 (0)	1 (0.8)	1 (1.3)	0.4 (0 - 1.2)
Partially controlled	24 (19.5)	22 (24.7)	43 (33.6)	18 (22.5)	25.3 (20.1 - 30.6)
Not controlled	98 (79.7)	67 (75.3)	84 (65.6)	61 (76.3)	74.3 (69.0 - 79.5)
**Pattern of symptoms - n (%)**					
Persistent	100 (81.3)	76 (85.4)	91 (71.1)	63 (78.8)	77.9 (72.9 - 82.9)
Intermittent	23 (18.7)	13 (14.6)	37 (28.9)	17 (21.3)	22.1 (17.1 - 27.1)
**Recommended treatment** - n (%)					
Leukotriene receptor antagonists (LTRA)	-	2 (2.3)	1 (0.8)	1 (1.3)	1.5 (0, 3.1)
Inhaled corticosteroids (ICS)	97 (78.9)	70 (78.7)	75 (58.6)	50 (62.5)	64.6 (58.8, 70.3)
ICS and long-acting β2-agonists (LABA)	6 (4.9)	4 (4.5)	30 (23.4)	14 (17.5)	16.7 (12.2, 21.2)
ICS with LTRA	9 (7.3)	8 (9.0)	5 (3.9)	2 (2.5)	4.6 (2.1 - 7.1)
Any asthma controller*	121 (98)	84 (94)	127 (99)	70 (88)	94.2 (91.3 - 97.0)
**Duration of controller - n (%)**					
Until better	3 (2.6)	1 (1.2)	6 (5.4)	2 (3.0)	2.5 (0, 5.0)
<1 month	7 (6.1)	3 (3.7)	10 (8.9)	2 (3.0)	3.9 (1.1 - 6.7)
1-2 month	8 (7.0)	2 (2.5)	12 (10.7)	3 (4.5)	5.4 (2.4, 8.4)
Long-term therapy †	97 (84.3)	77 (95.1)	84 (75.0)	60 (89.6)	86.0 (81.4, 90.6)
**Treatment objectives - n (%)**					
Treat current exacerbation	102 (82.1)	19 (21.3)	113 (88.3)	17 (21.5)	53.7 (47.7 - 59.7)
Improve short-term asthma control	72 (58.5)	38 (42.7)	76 (59.4)	24 (30.4)	45.4 (39.5 - 51.4)
Prevent future exacerbation	98 (79.7)	66 (74.2)	85 (66.4)	55 (68.8)	71.4 (65.9 - 76.8)
Improve long-term asthma control	97 (78.9)	82 (92.1)	88 (68.8)	73 (92.4)	84.0 (79.6 - 88.5)

* Asthma controllers include ICS, LTRA combination of ICS and long-acting ß2-agonists (ICS/LABA), theophylline and any combination thereof). Of note, theophylline was not selected by any participant.

† Long-term therapy was defined as prescribed for at least 3 months or until the patient sees this treating physician, the number o[illegible]

‡ Proportion adjusted for the stratified sampling of physicians by specialty.

Table E4. Beliefs about capabilities and consequences regarding the prescription of long-term asthma controller

	Child		Adult		
	Acute exacerbation *(Vignette 1)* (N=123) *	1 month after exacerbation *(Vignette 2)* (N=89) *	Acute exacerbation *(ignette3)* (N=128) *	1 month after exacerbation *(Vignette 4)* (N=80) *	Adjusted proportion (95% CI) (N=420) †
**Level of comfort for: ‡**					
Diagnosing asthma In:					
Preschoolers	4.0 (3.0, 5.0)	4.0 (3.0, 5.0)	2.0 (1.0, 3.0)	2.0 (1.0, 4.0)	1.9 (1.6, 2.1)
School-aged children	4.0 (4.0, 5.0)	4.0 (4.0, 5.0)	3.0 (2.0, 4.0)	4.0 (2.5, 4.0)	3.2 (3.0, 3.4)
Adults	3.0 (0, 4.0)	4.0 (1.0, 4.0)	4.0 (4.0, 5.0)	4.0 (4.0, 5.0)	3.6 (3.4, 3.8)
Individuals who smoke	3.0 (0, 4.0)	3.0 (1.0, 4.0)	4.0 (3.0, 4.0)	4.0 (3.0, 4.0)	3.3 (3.1, 3.5)
Distinguishing intermittent from persistent asthma					
Without pulmonary function tests	4.0 (3.0, 4.0)	4.0 (3.0, 4.0)	3.0 (2.0, 4.0)	3.0 (2.0, 4.0)	2.6 (2.4, 2.8)
Using pulmonary function tests	4.0 (3.0, 5.0)	4.0 (4.0, 5.0)	4.0 (3.0, 4.0)	4.0 (3.0, 5.0)	3.5 (3.3, 3.7)
Assessing the level of asthma control	4.0 (4.0, 5.0)	4.0 (4.0, 5.0)	4.0 (3.0, 4.0)	4.0 (4.0, 4.0)	3.4 (3.2, 3.6)
Initiating long-term inhaled corticosteroids (ICS)	4.0 (3.0, 5.0)	4.0 (4.0, 5.0)	4.0 (3.0, 4.0)	4.0 (4.0, 5.0)	3.5 (3.4, 3.7)
Making environmenttal recommendations	5.0 (4.0, 5.0)	5.0 (4.0, 5.0)	4.0 (4.0, 5.0)	4.0 (4.0, 5.0)	3.8 (3.5, 4.0)
Anticipating and answering patient's concerns	4.0 (4.0, 5.0)	4.0 (3.0, 5.0)	4.0 (3.0, 4.0)	4.0 (3.0, 4.0)	3.2 (3.1, 3.5)
Motivating patient to be more compliant	4.0 (3.0, 5.0)	4.0 (4.0, 5.0)	4.0 (3.0, 4.0)	4.0 (4.0, 5.0)	3.5 (3.3, 3.6)
**Hesitation towards prescribing long-term ICS in: ∫**					
Preschool-aged children	2.0 (1.0, 3.0)	2.0 (1.0, 3.0)	3.0 (2.0, 4.0)	3.0 (2.0, 4.0)	2.2 (1.9, 2.5)
School-aged children	1.0 (0, 2.0)	1.0 (0, 2.0)	2.0 (1.0, 4.0)	2.0 (1.0, 3.0)	2.4 (1.1, 1.6)
Adults-non smokers	1.0 (1.0, 3.0)	1.0 (0, 2.0)	1.0 (0, 2.0)	0 (0, 1.0)	0.1 (0, 0.2)
Adults - smokers	1.0 (0, 3.0)	1.0 (0, 2.0)	1.0 (0, 1.0)	0 (0, 1.0)	0.2 (0.1, 0.3)
Individuals with intermittent ashma	3.0 (1.0, 3.0)	2.0 (1.0, 3.0)	2.0 (1.0, 3.0)	2.0 (1.0, 3.0)	1.7 (1.4, 2.0)
Indiduals with mild asthma	3.0 (2.0, 4.0)	2.0 (1.0, 3.0)	2.0 (1.0, 4.0)	2.0 (1.0, 3.0)	1.8 (1.5, 2.0)
Individuals discharged following a visit	2.0 (0, 3.0)	1.0 (0, 2.0)	1.0 (0, 3.0)	1.0 (0, 1.0)	0.8 (0.5, 0.7)
Individuals discharged following a hospitalisation	1.0 (0, 2.0)	0 (0, 1.0)	1.0 (0, 2.0)	0 (0, 1.0)	0 (-0.1, 0.2)

* Data for each vignette are displayed as median (25%, 75%)

† Proportion adjusted for the stratified sampling of physicians by specialty, that is, weighting responses to reflect the distribution of physicians in the Province of Quebec using weights of 91.0% for family physicians, 7.6% for pediatricians, and 1.4% for emergency physicians.

‡ Likert scale of 0 (Not comfortable at all) to 5 (Very comfortable)

∫ Likert scale of 0 (No hesitation) to 5 (Considerable hesitation)

¶ Likert scale of 0 (Strongly disagree) to 5 (Strongly agree)

|| A paramedical healthcare professionnal includes nurses, certified asthma educators, pharmacists and respiratory technicians.

Table E5. Multivariate analysis of intention of prescribing long-term asthma controller *

	Intenders ¶ (N = 338)	Non Intenders ¶ (N = 82)	All cases	Pediatric Case Vignettes	Adult Case Vignettes
			Odd Ratios ∫ (95% CI)	Odd Ratios ∫ (95% CI)	Odd Ratios ∫ (95% CI)
**Types of symptoms** † - n (%)					
Persistent	281 (83.1)	49 (59.8)	3.57 (1.94, 6.54)	3.96 (1.57, 10.01)	2.98 (1.38, 6.45)
**Treatment objective** † - n (%)					
Improving long-term control	298 (88.4)	42 (51.2)	7.50 (3.59, 15.69)	4.45 (1.39, 14,21)	11.09 (3.93, 31.30)
**Hesitation in prescribing long-term ICS** ‡ - median (25%, 75**%)**					
In school-aged children	1.0 (0, 3.0)	3.0 (0, 4.0)	0.63 (0.50, 1.74)	0.60 (0.43, 0.83)	
In adult non-smokers	0 (0, 1.0)	1.0 (0, 3.0)			0.64 (0.46,0.88)
**Specialty - n (%)**					
Pediatrician	104(30.8)	11(13.4)	0.82 (0.34, 1.74)	0.95 (0.41, 2.22)	
Emergency Physician	31 (9.2)	25 (30.5)	0.59 (0.31, 0.11)	1.22 (0.38, 3.85)	0.44 (0.19, 0.95)
Family physician	203 (60.1)	46 (56.1)	1	1	1

Blank cells indicate that the variable was not statistically significant.

* This multivariate analysis was conducted without offering as a candidate variable 'physicians's reported comfort level in initiating long-term ICS.'

¶ Physicians who reported prescribing long-term ICS to the patient in their selected vignette were considered 'Intenders' in contrast to their counterparts considered 'Non Intenders.'

† Regarding the patient in their selected clinical vignette

‡ On a Likert scale of 0 (No hesitation) to 5 (Considerable hesitation)

∫ Odds ratio adjusted for speciality

Figure E1

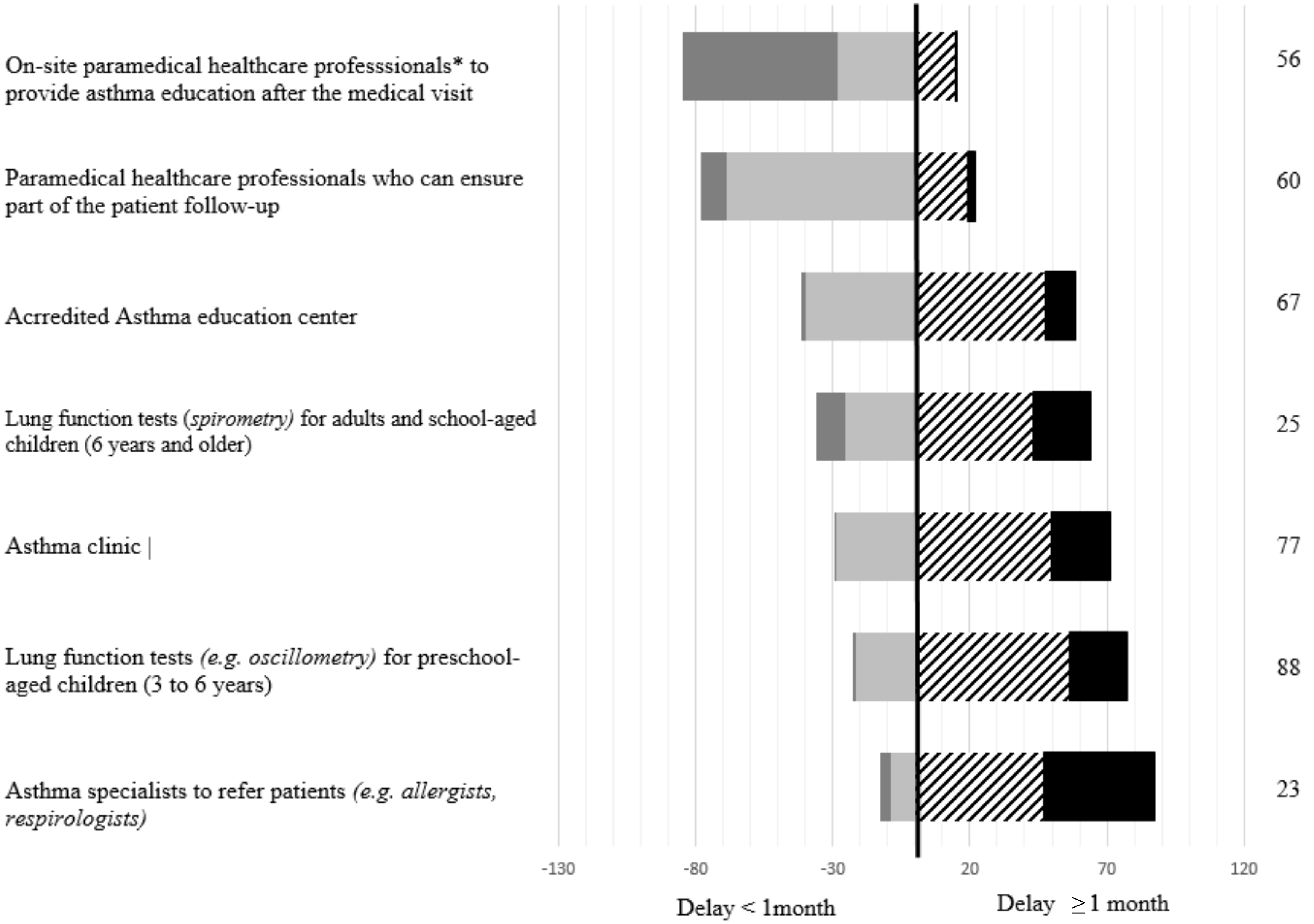

On-site paramedical healthcare professsionals* to provide asthma education after the medical visit
Paramedical healthcare professionals who can ensure part of the patient follow-up
Acrredited Asthma education center
Lung function tests (spirometry) for adults and school-aged children (6 years and older)
Asthma clinic |
Lung function tests (e.g. oscillometry) for preschool-aged children (3 to 6 years)
Asthma specialists to refer patients (e.g. allergists, respirologists)
56
60
67
25
77
88
23
-130
-80
-30
20
70
120
Delay < 1month
Delay ≥1 month

Legend

This histogram depicts the physicians' percent endorsement, adjusted for sampling fraction, of the reported delay to access each resource indicated on a 4-point Likert-like scale ranging from same day (dark gray), <1 month (light grey), 1-3 month (diagonal grey bars), and ≥4 month (black bars). The proportion of participants who unable to answer because the resource was not available or they did no know the expected delay is displayed in the right-sided column. * Paramedical healthcare professionals include nurses, certified asthma educators, pharmacists, and respiratory technicians. | An asthma clinic includes asthma specialists and paramedical healthcare professionals and in which patients with asthma are grouped in a specific time window.
